# Marker genes as predictors of shared genomic function

**DOI:** 10.1186/s12864-019-5641-1

**Published:** 2019-04-04

**Authors:** Joseph L. Sevigny, Derek Rothenheber, Krystalle Sharlyn Diaz, Ying Zhang, Kristin Agustsson, R. Daniel Bergeron, W. Kelley Thomas

**Affiliations:** 1 Molecular, Cellular, and Biomedical Sciences, University of New Hampshire, 46 College Rd, Rudman Hall, Durham, NH 03824 USA; 2 Department of Computer Science, University of New Hampshire, 33 Academic Way, Kingsbury Hall, Durham, NH 0324 USA; 3 Hubbard Center for Genome Studies, University of New Hampshire, 35 Colovos Rd, Gregg Hall, Durham, NH 03824 USA

**Keywords:** Metabarcoding, Metagenomics, 16S rRNA, Marker gene, Amplicon, Functional capacity, Comparative genomics

## Abstract

**Background:**

Although high-throughput marker gene studies provide valuable insight into the diversity and relative abundance of taxa in microbial communities, they do not provide direct measures of their functional capacity. Recently, scientists have shown a general desire to predict functional profiles of microbial communities based on phylogenetic identification inferred from marker genes, and recent tools have been developed to link the two. However, to date, no large-scale examination has quantified the correlation between the marker gene based taxonomic identity and protein coding gene conservation. Here we utilize 4872 representative prokaryotic genomes from NCBI to investigate the relationship between marker gene identity and shared protein coding gene content.

**Results:**

Even at 99–100% marker gene identity, genomes share on average less than 75% of their protein coding gene content. This occurs regardless of the marker gene(s) used: V4 region of the 16S rRNA, complete 16S rRNA*,* or single copy orthologs through a multi-locus sequence analysis. An important aspect related to this observation is the intra-organism variation of 16S copies from a single genome. Although the majority of 16S copies were found to have high sequence similarity (> 99%), several genomes contained copies that were highly diverged (< 97% identity).

**Conclusions:**

This is the largest comparison between marker gene similarity and shared protein coding gene content to date. The study highlights the limitations of inferring a microbial community’s functions based on marker gene phylogeny. The data presented expands upon the results of previous studies that examined one or few bacterial species and supports the hypothesis that 16S rRNA and other marker genes cannot be directly used to fully predict the functional potential of a bacterial community.

**Electronic supplementary material:**

The online version of this article (10.1186/s12864-019-5641-1) contains supplementary material, which is available to authorized users.

## Background

Characterizing the diversity, abundance, and functional capacity of microbial communities has remained an important but difficult task for scientists. Current next-generation sequencing studies typically employ either full metagenome analysis, in which the entire genomic content of a community is sequenced, or marker gene analysis (also known as amplicon-based sequencing or metabarcoding) where individual genes, most often 16S rRNA, are targeted using amplification with conserved primers. While these amplicon-based studies provide valuable insight into the diversity and relative abundance of taxa within communities, they provide no direct insight into the function or genomic content of a community. Recently, there has been a surge in the desire to predict functional capacity based on taxonomic assignment from these amplicon studies. In fact, phylogeny has been used to infer the molecular functions of microbes in the past and in recent papers [1–3]**.** Tools such as PICRUSt [4] and Vikodak [5] have been created to link amplicon data to functional predictions. However, using phylogeny to predict functional content has two major limitations: it is largely dependent on database coverage [4], and it doesn’t consider inputs from the local ecology (environmental conditions, taxa abundance, phage presence, etc.) in shaping community functions [6–8]. While authors are quick to acknowledge the limitations of inferred phylogeny to predict functional content, the limitations still exist.

There are multiple lines of evidence that 16S rRNA is not an ideal marker for characterizing functional content [9–12]. One of the most well-known studies shows that three different strains of *Escherichia coli* (two pathogenic and one non-pathogenic) share less than 40% of their gene products, even though their 16S sequences are identical [13]. Recent papers have also shown this heterogeneric relationship at the strain level with *Roseobacter spp.* and *Microbacterium spp.* [14, 15]. Thus, although at some level we already know the answer to this question, to date we have found no large-scale analysis to quantify the correlation between phylogenetic gene identity and functional capacity. An analysis of a broader spectrum of genomes has the potential to explore the more general limits of phylogenetic markers, such as 16S rRNA, to predict community function.

This study aims to survey and quantify the variability of the 16S rRNA gene and select conserved single-copy ortholog genes (housekeeping genes) to examine its relationship with shared gene content. For this relationship, we hypothesized that the correlation would follow two general rates of change. (1) There is an unpredictable proportion of the genome typically acquired by horizontal gene transfer (conjunction, transformation, and transduction) [16], this proportion of the genome is independent of a phylogenic timescale, and results in an initial decrease of mean shared gene content between phylogenetically identical organisms. These events are likely ‘random’ or environmentally driven making accurate predictions impossible. (2) The remaining proportion of the genome consists of genes associated with ‘core’ function, and thus are conserved across phylogenetically related organisms. As phylogenetic distance increases there is a proportional decrease in shared gene content, likely due to slow gene loss, pseudogenes, and differences in genomic architecture [17, 18]. Furthermore, we hypothesize that separate lineages/clades experience different rates of change, with respect to the adaptive and core genome. That is, certain lineages, such as *E. coli* and *Vibrio spp.*, are more prone to and efficient in laterally transferring DNA, this results in a large pool of genes that are unshared between phylogenetically related organisms [19, 20]. On the other hand, different lineages exhibit more of a genomic static state and result in a higher percentage of shared genes [21].

**Fig. 1:**
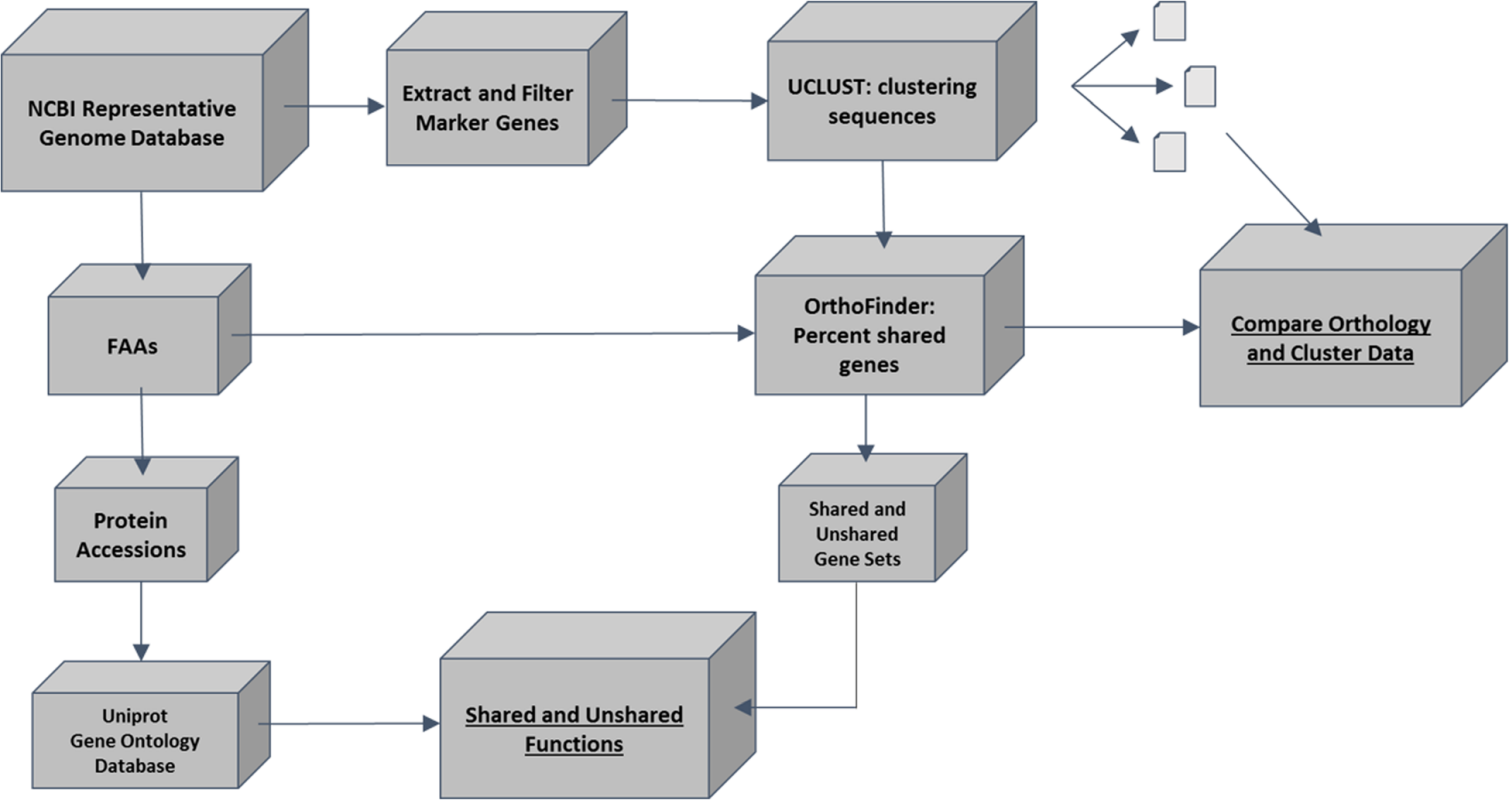
Workflow of data analysis. The workflow for analysis starts at the upper left box “NCBI Representative Genome Database” and follows two majors tracks. The first leads to a comparison between bacterial genome orthology (determined by Orthofinder) and marker gene sequence cluster groups (determined by UCLUST). The second path utilizes the protein-coding gene sets to determine which functions are shared or unshared across the bacterial genomes. Arrows correspond to the movement of data through the pipeline

To test these hypotheses, we conducted a comparative genomic study using 4872 well-annotated prokaryotic reference genomes from the publicly available RefSeq database on NCBI (Fig. 1). Utilizing these genomes, we examined the relationship between 16S rRNA divergence and shared gene content on a large scale. We also examined the divergence of several concatenated single-copy orthologs to determine if they offer a means to combat potential limitations in using the 16S rRNA. Lastly, we conducted a large-scale comparison between shared and novel gene sets to investigate the shared and novel functions of recently diverged prokaryotic organisms. Our goal was to determine aspects of the functional profile that may remain unknown when assuming a high predictable correlation between 16S identity and organismal protein-coding gene content.

## Results

### Prokaryote genome dataset and gene extraction

**Fig. 2:**
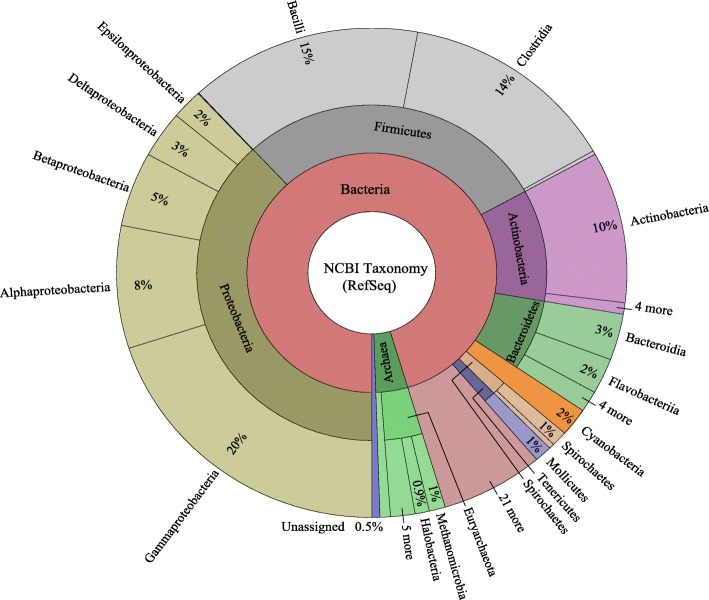
Taxonomic classifications of NCBI’s RefSeq representative prokaryotic genomes. A KronaTool map representing the relative taxonomic breakdown of the genomes used in this study. The inner circle represents genomes at the domain, the middle circle corresponds to phylum, and the outer circle represents data at the class level

A total of 4872 complete representative prokaryotic genomes are available through the NCBI ftp portal, spanning 28 of the 29 accepted bacterial phyla [22] and both classically accepted archaeal phyla, Crenarchaeota and Euryarcheaota [23]. Complete taxonomic distribution of the data, as constructed by Krona tools is shown in Fig. 2. See Additional file 1: Table S1 for a complete datasheet of all genome accessions and taxonomy used in this study. From this data, three amplicon datasets were generated: (1) Full-length 16S rRNA, including 10,072 sequences from 4773 genomes, ranging in length between 1001 and 1856 bps (μ = 1516.9, σ = 86.5, 2) The V4 region of the 16S rRNA, including 9710 sequences from 4426 genomes, ranging in length between 334 and 509 bps (μ = 412.3, σ = 4.6); and (3) Concatenated single-copy orthologs, including 3985 sequences (five genes), one for each genome, ranging in length between 6001 and 7434 bps (μ = 7001.9, σ = 376.5). In this multi-locus sequence analysis (MLSA) we chose five single-copy orthologs: 30S ribosomal proteins S12 and S15, GTPase Der, ATP-synthase delta, and CTP synthase, because of their uniform presence and nomenclature across the dataset.

### Intra organism 16S rRNA variation

**Fig. 3:**
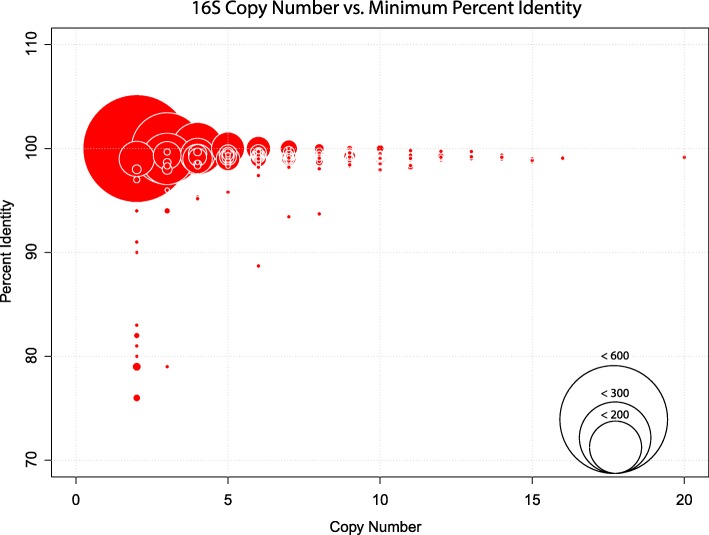
Relationships between intra-organism 16S rRNA copy number and the percent identity across copies. A scatter bubble plot represents the relationship between 16S rRNA copy number and the percent identity between those copies. The circle size corresponds to the number of bacterial genomes with the same percent identity and copy number

For genomes within this dataset, 16S rRNA copy number ranged from one (*n* = 2485) to twenty (*n* = 1, accession GCF_000686145), with an average of 2.3 copies per sample (σ = 2.1). The majority (99.1%) of the 16S rRNA copies with each genome examined have high sequence similarity (> 97%), however, a total of 38 genomes were found to have 16S rRNA copies that are less than 97% identical (Fig. 3). See Additional file 2: Table S2 for the full datasheet of 16S rRNA copy statistics. While no significant relationship between copy number and minimum gene identity was observed (R^2^ = 0.013), all genomes with less than 97% intra-genomic 16S copy identity have less than nine copies of the gene.

Through sequence alignment validation of the low percent identity copies we found that 15 of the genomes contain one or more 16S rRNA copies with long stretches of unidentified nucleotides (represented by N’s sequence) or had large gaps within the alignment. For example, the genome GCF_000332335 has five copies of the 16S rRNA gene, four of which were nearly identical while one sequence was highly diverged and contained several stretches of unidentified nucleotides. In such cases, these highly diverged copies were not included in the analyses comparing 16S copy number and sequence variation.

### Sequence clustering

To explore the relationship between gene identity and shared gene content, we first clustered all marker gene sequences at intervals between 95 and 100%. This was completed separately for each of the three datasets (complete 16S, variable region of 16S, and MLSA). For the complete 16S rRNA and the V4 16S rRNA datasets, a large proportion of the 16S rRNA copies from the genomes clustered with 16S rRNA copies from different genomes at or above 95% sequence similarity (71 and 80% respectively), thus retaining a large number of comparisons for this analysis. Because only 8% of the MLSA dataset concatenated sequences clustered with sequences from other genomes at 95% or greater, we included further comparisons at 93.0–93.9% and 94.0–94.9%. At this range 520 marker gene sequences (13%) clustered into groups with two or more unique genomes.

**Fig. 4:**
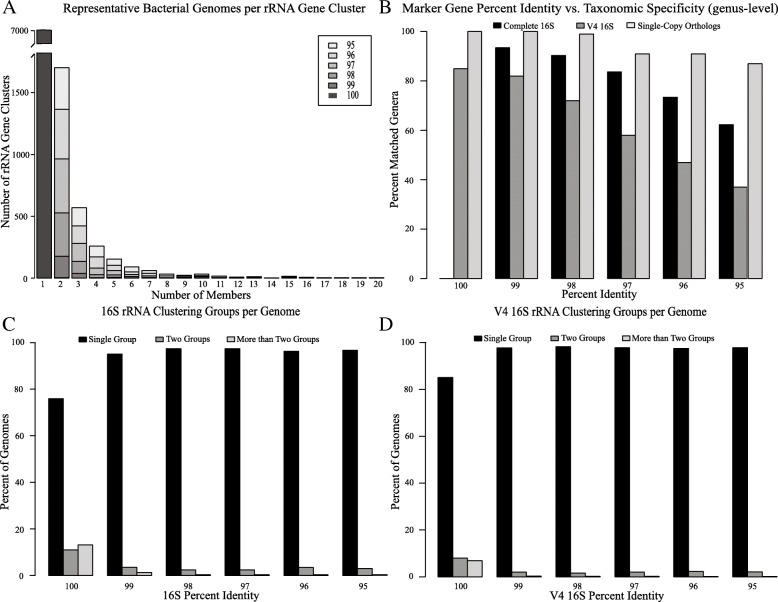
16S rRNA clustering statistics. **a** The relationship between the number of 16S rRNA clustering groups and the number of bacterial genomes represented in each cluster at various percent identity thresholds. **b** Taxonomic resolution (genus level) based on clustered marker genes for each of the three amplicon datasets. **c** and **d** The percentage of genomes whose 16S rRNA genes clustered into one, two, or greater than two different clustering groups for the 16S rRNA and V4 16S rRNA datasets respectively

A representative graph depicting the sequence clustering of the complete 16S rRNA dataset for each percent identity group can be seen in Fig. 4 (a). The y-axis depicts the total number of 16S rRNA clustering groups and the x-axis depicts the total number of unique genomes (as represented by their 16S rRNA sequence) found within the respective clustering group. A similar trend was observed for each dataset. As the percent identity of the marker genes decreases there is an increase in marker gene clusters that include two or more genomes.

### Intra-organism 16S rRNA copies and sequence clustering

As previously shown, many of the genomes in this dataset have low sequence similarity across their 16S rRNA copies. To investigate how this affected sequence clustering we tracked the 16S rRNA copies for each genome across the clustering groups. Figure 4(c) and (d) depict the percentage of genomes whose 16S rRNA copies are found in one, two, or greater than two different 16S rRNA clustering groups for the complete 16S and V4 16S dataset respectively. As shown, the majority of the 16S rRNA copies from a single genome cluster into a single group, however, some are effectively divided and grouped with 16S rRNA copies from a different genome. This is true regardless of the sequence identity threshold used.

### Marker gene sequence identity and taxonomic identification

Determining proper phylogenetic identification is often an important step in metabarcoding analyses, we therefore examined the taxonomic relationships between genomes at various marker gene sequence identity thresholds. As shown in Fig. 4 (b), the MLSA-like approach shows the highest correlation between percent identity and taxonomic matches at the genus level. Even at 98% sequence similarity, 99% of the genomes cluster into groups with their respective genera. By contrast, genomes with 100% V4 16S rRNA identity show only 85% taxonomic matches at the genus level and 58% taxonomic matches at the genus level by 97% sequence identity.

### Percent shared genes vs. marker gene similarity

**Fig. 5:**
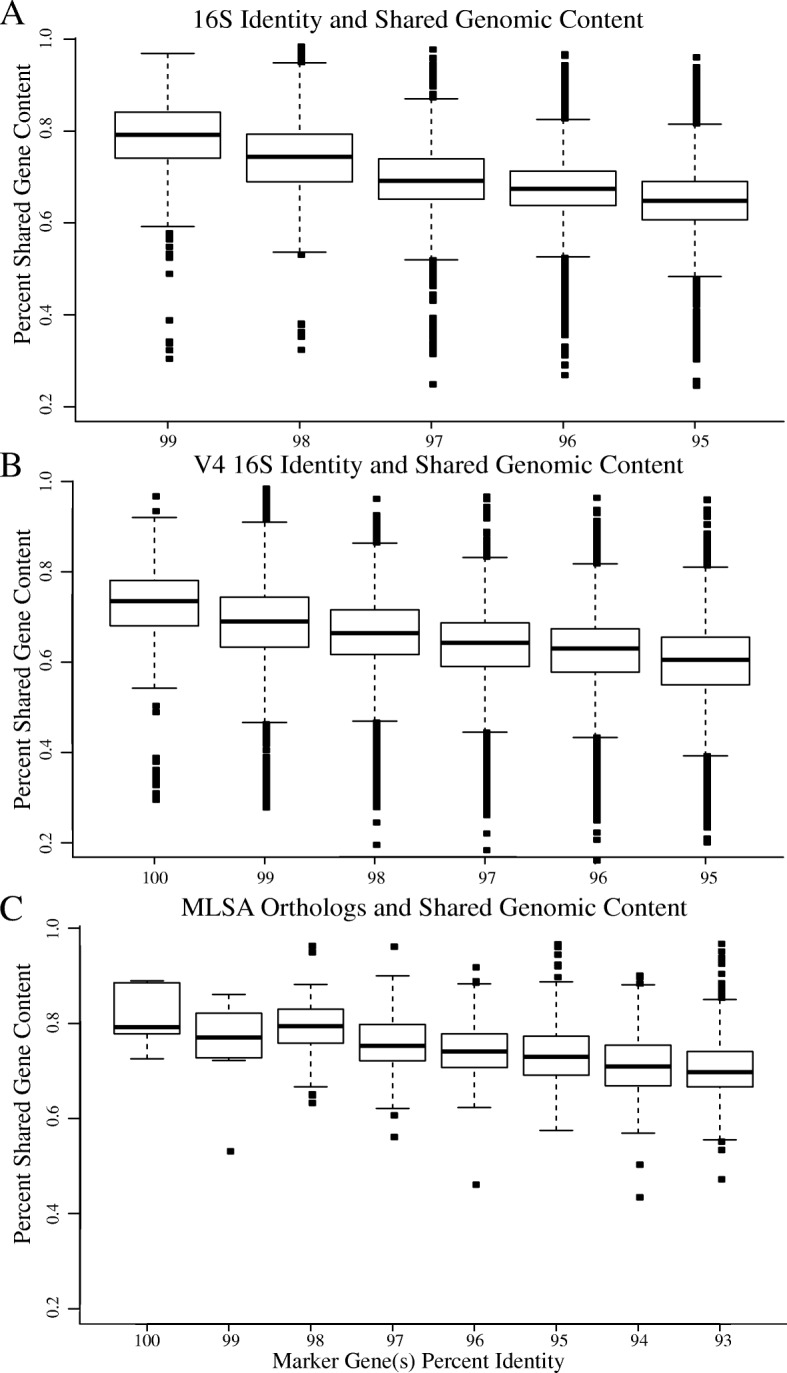
Phylogenetic marker(s) and single-copy ortholog(s) relationship to shared gene content. Shown are box and whisker plots depicting the percentage of shared genes between genomes clustered at various percent identity intervals: (**a**) 16S rRNA, (**b**) V4 16S rRNA, (**c**) Five-concatenated MLSA orthologs. Boxplots show the first and third quartile (bottom and top lines of the box), the median (middle line of the box), and the smallest and largest data-points excluding outliers (bottom and top whiskers). Data-points outside the whiskers correspond to outliers

After clustering marker gene sequences into sequence similarity intervals, pairwise comparisons of protein coding gene content were completed for each genome using Orthofinder. Percent shared genes is defined here as the ratio between the number of genes matched among two genomes and the total number of genes present in both. Figure 5 depicts the relationship between similarity cutoff values and the percent shared gene content for the three different marker gene data sets. Among all comparisons, the percent shared genes range from 24.6 to 98.4% and results show an initial decrease in shared genes between organisms whose marker genes cluster at 100% or 99%. At the highest percent identity interval, the arithmetic means for each marker gene dataset are as follows; 78% shared gene content at 99% 16S rRNA identity, 72% shared gene content at 100% V4 16S rRNA sequence identity, and 83% shared gene content at 100% MLSA sequence identity. While all datasets show a similar trend, the decrease in average shared gene content between cluster groups is highest in clustering done via the V4 16S rRNA (Fig. 5a) and lowest in the single-copy ortholog dataset (Fig. 5c). See Additional file 3: Table S3 for data used in construction of Fig. 5.

**Fig. 6:**
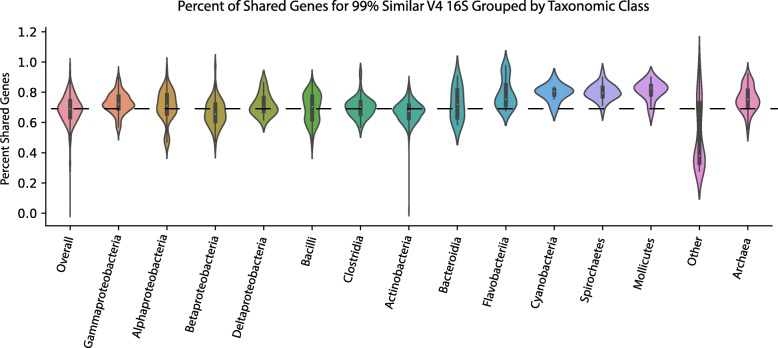
Relationship between 99% similar V4 16S rRNA and shared gene content across select microbial lineages. Violin plots representing the distribution of phylogenetically identical organisms (99% V4 16S rRNA) across select microbial lineages and the percentage of shared gene content. The dotted black line corresponds to the mean shared gene content of the entire dataset and the width of the violin represents the relative concentration of data (i.e. wider regions contain more data points)

To expand upon these findings, we wanted to determine if there are certain groups of bacterial lineages where the relationship between marker gene identity and shared genome composition is higher or lower than the combined dataset (Fig. 6). We examined this in the V4 16S dataset at 99% sequence similarity but expect similar trends for other marker genes. Based on a Kruskal-Wallis test with a Dunn’s multiple comparisons and Bonferroni correction the data shows that Spirochetes, Gammaproteobacteria, Cyanobacteria, Mollicutes, Archaea, and Flavobacteriia have a higher mean percent of shared genes (*p* < 0.05). The group termed “Other” is comprised of highly similar 16S sequences that span different bacterial classes. As expected, these comparisons contain a significantly lower number of shared genes (Fig. 6; *p* < 0.05). Other classes of bacteria, like Bacilli, Clostridia, and Alphaproteobacteria contain similar amounts of shared genes when compared to the overall dataset.

To further validate these findings and test if the unshared genes may have been misannotated or if their functionality was lost due to rapid evolution, we subjected unshared genes to a tBLASTn search against complete genomes in the clustering group rather than their associated protein FASTA file. We found that most of these genes (μ = 87.4%, σ = 0.3) are not found in the closely related genome at > 70% identity and only 1.8% on average are found at greater than 95% identity (Additional file 4: Table S4).

### Shared and novel functions

Data presented thus far shows genomes clustered via identical or near identical 16S or single-copy orthologs share, on average, only 72–83% of protein-coding gene content. To determine if specific functions are more likely to appear in the shared or unshared across closely related bacterial genomes data sets, we analyzed the gene ontology (GO) of the matched and unmatched proteins identified from Orthofinder. We chose to focus on genomes whose V4 16S rRNA clustered at 99% sequence similarity or greater. This dataset consists of 6,324,117 protein accessions and 3515 total genome-genome comparisons. Of these accessions, 3,791,226 are found in the UniprotKB databases with a total of 2,803,829 containing gene ontology metadata. Results show 1794 GO terms significantly more likely to be shared, and 1119 GO terms more likely to be novel (unshared) (*p*-value < 0.01) (Additional file 5: Table S5). The top five significant shared and novel functions for each of the GO groups (biological process, molecular function, and cellular component) are shown in Table 1.

**Table 1:** Significant shared and unshared gene ontology terms between phylogenetically identical organisms (99% V4 16S rRNA)

Ontology	GO.ID	Term	Count-shared	Count-unshared	*P*-value
Molecular Function					
*unshared*	GO:0004803	transposase activity	4591	8641	< 1e-30
	GO:0003964	RNA-directed DNA polymerase …	165	288	< 1e-30
	GO:0097351	toxin-antitoxin pair type II bind …	72	274	< 1e-30
	GO:0090729	toxin activity	357	915	< 1e-30
	GO:0009036	type II site-specific deoxyribon …	24	180	< 1e-30
*shared*	GO:0019843	rRNA binding	42,179	808	< 1e-30
	GO:0046872	metal ion binding	124,123	28,675	< 1e-30
	GO:0003735	structural constituent of ribos …	63,194	2123	< 1e-30
	GO:0003723	RNA binding	32,770	4032	< 1e-30
	GO:0000287	magnesium ion binding	50,000	8454	< 1e-30
Biological Function					
*unshared*	GO:0032196	transposition	1435	1887	< 1e-30
	GO:0045927	positive regulation of growth	69	327	< 1e-30
	GO:0045926	negative regulation of growth	86	338	< 1e-30
	GO:0051607	defense response to virus	235	756	< 1e-30
	GO:0043571	maintenance of CRISPR repeat …	162	560	< 1e-30
*shared*	GO:0006412	translation	70,775	2978	< 1e-30
	GO:0071555	cell wall organization	18,821	1788	< 1e-30
	GO:0006457	protein folding	11,000	826	< 1e-30
	GO:0009252	peptidoglycan biosynthetic proc. …	16,336	977	< 1e-30
	GO:0008360	regulation of cell shape	17,552	1049	< 1e-30
Cellular Component					
*unshared*	GO:0012506	vesicle membrane	143	220	< 1e-30
	GO:0009341	beta-galactosidase complex	487	567	< 1e-30
	GO:0031469	polyhedral organelle	37	149	< 1e-30
	GO:0008305	integrin complex	38	83	< 1e-30
	GO:0030077	plasma membrane light-harv …	68	147	< 1e-30
*shared*	GO:0015934	large ribosomal subunit	8067	252	< 1e-30
	GO:0005623	cell	15,927	4518	< 1e-30
	GO:0005886	plasma membrane	157,863	45,460	< 1e-30
	GO:0015935	small ribosomal subunit	7833	98	< 1e-30
	GO:0005737	cytoplasm	248,487	26,478	< 1e-30

The top five GO terms (ordered by p-value) for each of the three broad categories of ontology: biological process, molecular function, and cellular component. ‘Count-shared’ and ‘Count-unshared’ refer to the number of times that particular GO term was found to be shared or unshared in the genome wide protein-coding content comparisons with Orthofinder. For an expanded summary of significant GO terms, see Table S5 in Additional file 5.

## Discussion

### Dynamic genome evolution

The current study quantifies the functional evolution of microbial genomes by describing the relationship between marker gene identity and shared protein-coding gene content. Results show that prokaryotic genomes exhibit a dynamic rate of evolutionary change. Although most of the genome mimics a rate of change following marker gene divergence, on average, 22–28% of the genome is independent of phylogenetic identity (Fig. 5). This dynamic nature can be explained by three general phenomena: 1) large introduction of non-native DNA from events like horizontal gene transfer; 2) gene deletion/loss of function; and 3) significant differences between genes in their evolutionary change. However, when we compared the unshared genes of genomes with high marker gene sequence similarity, we found that most of these genes have no matches even at low sequence identity thresholds, indicating that different rates of evolutionary change do not contribute significantly to genomic divergence in the tested genomes. We therefore propose that gene deletion, along with large introduction of non-native DNA, are more probable explanations for the results shown here. These phenomena are largely dependent on the organism’s environment, resulting in a portion of the genome that is dependent on microbial niche, selective pressures, and environmental conditions [24–28].

### Choice of marker gene

We analyzed the complete 16S rRNA, the variable portion of the 16S rRNA, and various single-copy orthologs through an MLSA-like approach. We expected that the greater resolution by the complete 16S dataset and MLSA approach would significantly dissect the observed rapid change in gene content, but it was only marginally improved. Although marginal, these results support the use of an MLSA approach to improve the resolving power between shared protein-coding gene content and percent identity. This style of analysis has been routinely used in genotyping pathogens, such as methicillin-resistant *Staphylococcus aureus* [29] or differentiating lineages or strains within a species [30, 31].

### Intra organism 16S variation and genome clustering

Evidence shown in both Fig. 3 and Fig. 4 (c and d) suggest that there is a subset of genomes with a few highly divergent copies of the 16S gene. While we observed that the majority of 16S copies within a genome have high sequence similarity (> 97%), many contain 16S copies with > 3% divergence, and similar findings have been reported in previous literature [32–34]. Furthermore, we observed that all genomes with greater than 3% divergence in 16S copies are genomes with less than nine total copies (Fig. 3). Although untested here, this phenomenon may be an artifact of the assembly process, where sequences may become biased toward a consensus when deduced based on highly covered reads/kmers, such as those from genes with a high copy number. Conversely, this may reflect a mechanism of conserved evolution where genomes with greater copy numbers avoid unwarranted gene sequence changes via redundancy.

Figure 4 depicts how these divergent copies clustered within UCLUST. In cases where 16S copies clustered into more than one group, some copies of the 16S are more like copies in a different genome than 16S copies within their own. In these cases, a single organism would be represented by several sequence variants and have a direct effect on functional prediction as well as affecting abundance estimations based on marker gene identity, even at the 97% species level criteria. Based on these observations a 97% criteria for clustering species is no more informative than 96% or 98%. Even so clustering and predicting OTUs or assigning species level taxonomy based on 97% identity is practical and informative in most cases.

The field is moving away from using OTU sequence clustering for amplicon analyses and has begun to use exact sequence variants instead (i.e., 100% sequence similarity clustering after error-correction) [35]. The results shown here mainly support this transition and expand upon the problems of using the typical 97% OTU clustering for variant identification. For example, it is likely that OTUs are unnecessarily reducing our resolution by potentially grouping different genera into a single OTU (Fig. 5b). However, using exact sequence variants (or 100% OTU clustering) does not offer a means to combat the intra-organism 16S rRNA variation that often exists within an organism’s genome (Figs. 4 and 5). This observation remains regardless of whether OTU clusters or exact sequence variants are used and remains a limitation with amplicon studies.

### Functional analysis

The purpose of the GO enrichment analysis was to determine if the functions that change rapidly are unique and predictable. Additionally, we wanted to identify what functions are lost when a close correlation between marker gene identity and overall functional capacity is assumed. We found that although most gene ontology terms are shared across the genome dataset, many important and unique functions are significantly more prevalent in the novel/unshared gene sets (Table 1). Key functions such as ‘transposase activity’ (molecular function), ‘transposition’ (biological processes), and ‘vesicle membrane’ (cellular component) are the top hits across GO terms within this novel dataset. These processes may be related to horizontal gene transfer and represent key functions that could mediate microbial niche adaptation. Furthermore, many functions related to metabolic processes, such as ‘glucosidase activity’ or ‘fucose metabolic processes’, which may also be crucial to a specific environmental niche, are found in the unshared datasets**.** From the thousands of pairwise comparisons, we found that these functions are more likely to be found in unshared gene datasets. Within the shared datasets we observed GO terms such as ‘DNA repair’, ‘DNA binding, and ‘integral component of the plasma membrane’. These are essential components that are necessary for a microbe to function, regardless of environment.

These findings related to the novel/unshared PCG functions are expected and similar to the observation of a core and accessory genome within microbes and supports the pan-genome concept, which is the collection of shared genomic resources that varies across environments [36]. When scientists study the microbial community of a novel environment, they are often interested in how that community functions and differs from other known communities. By grouping species based on marker gene(s) sequence similarity and predicting functional content, we miss much of the novel functions or overestimate the functional capacity. This prevents thorough comparison of two communities and potentially hinders the discovery of novel functions, an aspect that may have motivated such a study in the first place.

### Dataset and potential bias

The RefSeq representative prokaryotic genome database contains a large and diverse representation of major bacterial taxa for a comprehensive microbial dataset. All included genomes underwent a consistent annotation pipeline and nearly all protein-coding genes are linked to RefSeq GenBank files, so annotations and gene functions can be determined programmatically in an efficient manner. However, because many of the genomes available are biased towards biomedically and clinically relevant taxa, we anticipate some level of bias in the functional content of these organisms.

### Implications

Authors of programs aimed at inferring functional content from amplicon data are quick to acknowledge the limitations that are expanded upon here. PICRUSt does provide a QC metric, Nearest Sequenced Taxon Index (NSTI), which can help elucidate the limitation of database coverage and aid in interpretation of data. However, databases such as GenBank are severely biased towards easily culturable bacteria, like *Proteobacteria*, which comprises of 46% of the genomes sequenced [37], leaving unculturable bacteria vastly uncharacterized. Depending on the environment sequenced, this could lead to a majority of bacterial functions being predicted from distantly related genomes [38]. Thus, programs such as PICRUSt and Vikodak promote a potentially misguided idea that the presence of certain organisms corresponds to what functions they should be carrying out. At best such programs can present hypotheses to be tested.

## Conclusions

The central hypotheses in this study address the relationship between marker gene identity and protein coding gene content. We observed with overwhelming evidence that even phylogenetically identical organisms do not share substantial proportions of their gene products, highlighting the gap between marker gene identity and protein-coding gene content. Specifically, we found that 22–28% of an organism’s functional capacity cannot be determined from marker gene(s) alone, even with MLSA. This is true even when analyzing 100% identical sequences, demonstrating the limitations of amplicon-based studies and their ability to characterize the functional capacity of microbial communities. Future studies using additional marker genes or other variable portions of the 16S gene, along with environmental datasets, would build on the results presented here and further elucidate the dynamics of microbial evolution.

## Methods

### Prokaryote genomes and 16S extraction

Prokaryotic genome and assembly accessions were identified from the NCBI representative genome report file. Corresponding genome/assembly FASTA, general feature format (GFF), amino acid FASTA (FAA), and GenBank feature format (GBFF) files were then downloaded via the NCBI ftp server ([39], release 75). Taxonomic information for each sample was determined from the README file within the ftp repository. Visualization of taxonomic information was completed with Krona tools v2.2 [40]. For each sample, a Python script was used to extract the 16S rRNA gene sequences from the genome assembly FASTA file. Gene identifications, direction, start, and stop locations were obtained directly from the corresponding GFF files. Sequences less than 1000 bps in length were removed from the dataset and not included in subsequent steps. For each genome, 16S copy number, sequence lengths, and intra-organism gene variation statistics were calculated. For genomes with two or more 16S sequences, average and pairwise percent identity between 16S rRNA copies was determined using the T-Coffee v11.0 seq_reformat utility sim_idscore [41]. For genomes with two or more 16S rRNA copies that are less than 99.9% identical an alignment was constructed using Muscle v3.8.31 [42] and examined to validate the sequences and annotations.

### Extraction of the 16S variable region

An additional parallel dataset consisting of only the V4 variable region of each 16S rRNA gene was also constructed. In this approach the 16S variable region were extracted from each 16S rRNA sequence bioinformatically using a pair of primers commonly used for amplicon studies, the 515f (GTGYCAGCMGCCGCGGTAA) forward primer and 926r (CCGYCAATTYMTTTRAGTTT) reverse primer.

### Single copy orthologs extraction and concatenation

We constructed a third and final dataset consisting of concatenated single-copy orthologs to test a multi-locus sequence analysis (MLSA) like approach. Following the example of previous studies [43–45], single-copy orthologs present in at least 90% of bacterial species were identified using OrthoDB [29]. Out of the many potential genes identified, we chose five based on consistent annotation nomenclature and their presence as single copy genes across our dataset. For each organism, we extracted the five gene sequences from the genome assemblies and then concatenated them into a single sequence.

### Clustering gene sequences

For each of the three datasets (full-length 16S, V4 16S, and MLSA), we used UCLUST software v1.2.22q [46] to cluster the prokaryotic sequences into a set of clusters based upon sequence similarity. We clustered the sequences using identity thresholds of 95, 96, 97, 98, 99, and 100% to discern meaningful trends.

To investigate how the 16S rRNA gene copies from a single genome fell out into clustering groups, we examined the number of unique clustering groups per genome and identified any genomes whose rRNA copies were found in different clustering groups. In addition, we identified the number of unique genomes represented in each clustering group and their taxonomic assignments.

### Calculating percent shared genes

Next, we wanted to calculate the shared gene content between all genomes represented within the marker gene clustering groups using the program OrthoFinder v0.4, with default settings [47]. For validation of this method we subjected unmatched genes identified in Orthofinder to a tBLASTn search against the complete genomes of other members in the respective cluster. We recorded significant matches (e-value <1e-10) with a query coverage and percent identity greater than 70%.

The shared gene content comparisons were then linked back to the marker gene clustering groups obtained from UCLUST. Each comparison is only included in the highest percent identity group, effectively dividing the data into comparisons from 95.0–95.99, 96.0–96.99, 97.0–97.99, 98.0–98.99, 99.0–99.99, and 100% marker gene identity. Using R v2,14.2, we created box plots depicting shared content in relation to percent marker gene identity for each of the three datasets.

To determine if different lineages exhibit a higher or lower relationship between shared gene content and marker gene percent identity compared to the complete dataset, we split the V4 16S dataset into each of the major bacterial classes and completed the marker gene clustering and percent shared gene calculations outlined above. A Kruskal-Wallis test followed by a Dunn test for multiple comparisons with a Bonferroni correction was then completed to determine if the mean percent shared genes for each lineage was significantly different than the complete dataset.

### Determining shared and novel functions

To better understand the differences in shared and novel functions of closely related genomes, we examined all protein-coding genes from genomes whose V4 16S clustered together at 99% identity. First, the count of each protein accession within a matched or unmatched Orthofinder output file was determined across all comparisons. We linked accessions to gene ontology (GO) using the UniprotKB Swiss-Prot and TrEMBL databases (download date: May 01, 2016). Protein accessions and their respectively mapped GO terms were imported into the topGO software v3.8 [48]. For each gene ontology environment (molecular function, biological process, cellular component) enrichment of matched and unmatched GO’s were tested using Fisher’s exact test with the ‘weight01’ algorithm.

### Statistical analyses

All routine statistical analyses were performed in either Python v3.4 or R v2.14.2 with plottrix package [49].

## Additional files

Additional file 1: **Table S1.** The table provides the genome accessions and taxonomy for all reference sequences used in this study. (XLSX 169 kb)

Additional file 2: **Table S2.** Summary of 16S rRNA copy statistics for each bacterial genome. Included is the number of 16S rRNA copies, the minimum and maximum length among copies, the average length of copies, and the minimum and maximum percent identity among copies. (XLSX 93 kb)

Additional file 3: **Table S3.** A list showing the ratio of shared genes for each bacterial genome comparison. Data is included for each of the three datasets; Full-length 16S rRNA, V4 16S rRNA, and MLSA. (XLSX 1048 kb)

Additional file 4: **Table S4.** Summary of BLAST results comparing the unshared gene content of bacterial genomes whose V4 16s rRNA clustered at 99% identity. (XLSX 23 kb)

Additional file 5: **Table S5.** The table provides details of all significantly shared and unshared GO terms for bacterial genomes whose V4 16S rRNA sequences clustered at greater than 99% identity. (XLSX 158 kb)

## Abbreviations

16S rRNA: 16S Ribosomal RNA
BLAST: Basic Local Alignment Search Tool
*E. coli*: *Escherichia coli*
GO: Gene Ontology
MLSA: Multilocus Sequence Analysis
NCBI: National Center for Biotechnology Information
NSTI: Nearest Sequenced Taxon Index
OTU: Operational Taxonomic Unit
RefSeq: Reference Sequence Database
tBLASTn: Protein-Nucleotide 6-frame translation (BLAST)
